# Treatment Strategies for Infections With Shiga Toxin-Producing *Escherichia coli*

**DOI:** 10.3389/fcimb.2020.00169

**Published:** 2020-05-06

**Authors:** Sabrina Mühlen, Petra Dersch

**Affiliations:** ^1^Institute for Infectiology, University of Münster, Münster, Germany; ^2^German Center for Infection Research (DZIF), Associated Site University of Münster, Münster, Germany

**Keywords:** STEC, Shiga toxin, antibiotics, antibodies, vaccines

## Abstract

Infections with Shiga toxin-producing *Escherichia coli* (STEC) cause outbreaks of severe diarrheal disease in children and the elderly around the world. The severe complications associated with toxin production and release range from bloody diarrhea and hemorrhagic colitis to hemolytic-uremic syndrome, kidney failure, and neurological issues. As the use of antibiotics for treatment of the infection has long been controversial due to reports that antibiotics may increase the production of Shiga toxin, the recommended therapy today is mainly supportive. In recent years, a variety of alternative treatment approaches such as monoclonal antibodies or antisera directed against Shiga toxin, toxin receptor analogs, and several vaccination strategies have been developed and evaluated *in vitro* and in animal models. A few strategies have progressed to the clinical trial phase. Here, we review the current understanding of and the progress made in the development of treatment options against STEC infections and discuss their potential.

## Introduction

Shiga-toxin producing (enterohemorrhagic) *Escherichia coli* (STEC/EHEC) are a major cause of severe gastrointestinal disease in industrialized countries and a major public health problem with most frequent and severe infections linked to serotype O157:H7 (Kaper and O'Brien, 2014). The bacteria are commonly transmitted through ingestion of contaminated food such as undercooked meat, particularly beef products, cross-contaminated raw vegetables, sprouts, and seeds (Caprioli et al., 2014).

The resulting disease ranges in intensity from watery diarrhea or hemorrhagic colitis to the life-threatening hemolytic uremic syndrome (HUS) leading to kidney failure and neurological episodes (Nataro and Kaper, 1998). Upon ingestion, EHEC resides in the intestinal tract and adheres to the gut epithelium of the distal ileum and colon. Initial binding is promoted by fimbriae, which, in EHEC infections (e.g., by EHEC O157:H7, O126, O103, O45, O111, O121, O145), is followed by the injection of effector proteins (Esp proteins) via a filamentous type III secretion system (T3SS) (Donnenberg and Kaper, 1992; Garmendia et al., 2005; Gaytan et al., 2016). Injection of the translocated intimin receptor (Tir), which integrates into the host cell plasma membrane and interacts with the bacterial outer membrane protein intimin, initiates bacterial attachment to the host cell and effacement of the brush border microvilli. The interaction between intimin and Tir leads to intimate attachment of the bacteria and initiates actin polymerization and subsequent formation of attaching and effacing (A/E) lesions (Kenny et al., 1997). The genes encoding Tir, intimin, and the T3SS are localized on the chromosomal “locus of enterocyte effacement” (LEE) pathogenicity island. Notably, this island is missing from LEE-negative STEC and from the unusual HUS-inducing *E. coli* strain EAHEC of serotype O104:H4, which is responsible for the major outbreak in Germany and parts of Europe in 2011. This latter strain is similar to enteroaggregative *E. coli* (EAEC) (Bielaszewska et al., 2011; Mellmann et al., 2011).

While the HUS-inducing strains belong to a variety of *E. coli* pathovars, their main discerning trait is the production of at least one of two genetically distinct Shiga toxins, named Stx1 and Stx2. Four subtypes of Stx1 (Stx1a, Stx1c, Stx1d, Stx1e) and seven subtypes of Stx2 (Stx2a-g) have been identified, of which especially the Stx2 variants Stx2a and Stx2c are commonly associated with HUS development in humans (Melton-Celsa, 2014). Both types of Shiga toxins are AB_5_ toxins that bind to the glycosphingolipids globotriaosylceramide (Gb3, CD77) and, to a lesser extent, globotetraosylceramide (Gb4) (Legros et al., 2018), which are found on a variety of human cells, such as glomerular and brain endothelial cells. The Stx toxins result in the arrest of protein translation and, ultimately, cell death (Melton-Celsa, 2014). The systemic consequences of intoxication are vascular dysfunction and thrombus formation, which lead to HUS. The genes encoding for Stx are located in the late region of a lambdoid phage, which adds additional complications to treatment options. As several antibiotics, especially those belonging to the quinolone family were shown to be potent inducers of the bacterial SOS response, which initiates the production and release of phages from the bacteria, treatment of STEC infections with antibiotics is generally not advised (Kakoullis et al., 2019). To date, there are no protective measures or therapies against STEC infections. Current treatment of STEC infections is solely supportive and includes rehydration therapy, and, where necessary, dialysis. However, over the past years, new therapeutic approaches and novel, promising strategies to manage the infection and the ensuing disease have been developed. These are outlined in this review.

## Antibody Therapy

### Stx-Targeted Antibodies

**Figure 1:**
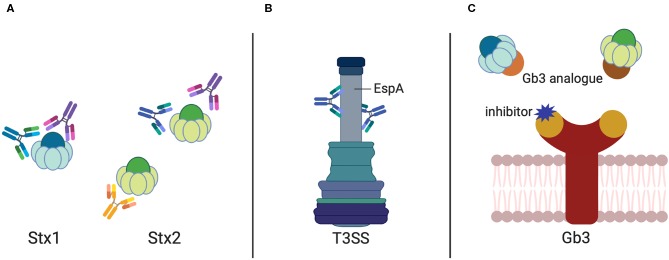
Antibodies and Gb3 analogs against STEC-induced diseases. Antibody targets in STEC treatment include **(A)** the Shiga toxins (Stx1 and Stx2) and **(B)** the sheath component EspA of the *E. coli* Type-3-Secretion System. **(C)** Analogs to the Stx receptor Gb3 harboring the Stx binding domains (given in brown and orange) sequestering Stx1 and Stx2. Inhibitors blocking the Stx binding site of the Gb3 receptor are illustrated in purple.

Antibodies are valuable therapeutics. As Stx-specific antibodies can completely neutralize the cytotoxicity of the toxin in cell culture and protect animals from developing Stx-induced symptoms when administered shortly after infection (Cheng et al., 2013), effective Stx-targeting antibodies are a suitable option for human therapy (Figure 1A).

Mohawk et al. (2010) investigated the ability of polyclonal Stx2-neutralizing antibodies from rabbits to protect mice from lethal infection with the Stx2a-producing *E. coli* O157:H7 strain 86-24. The administration of the rabbit serum did not reduce the initial colonization of mice with EHEC 86-24, but it decreased the bacterial burden after 3–5 days and increased animal survival.

Bovine colostrum preparations harboring high titers of Stx1 and Stx2 antibodies were used to treat mice infected with *E. coli* O157:H7. This efficiently inhibited bacterial attachment, colonization, and growth (Funatogawa et al., 2002). Although the frequency of stool excretion was reduced, the presence of the bacterial toxin was not notably affected (Kuribayashi et al., 2006; Seita et al., 2013). Furthermore, colostral IgG against Shiga toxin and bovine lactoferrin completely prevented lethality of *E. coli* O157:H7 in a weaned mouse model (Albanese et al., 2018). In addition, an early study in children showed that bovine colostrum is well-tolerated, reduced the frequency of loose stools, and eliminated bacterial infection (Huppertz et al., 1999).

Another interesting concept for this strategy is the production of recombinant antibodies or even secretory antibodies specific for Stx by transgenic plants (e.g., thale grass—*Arabidopsis thaliana*) (Nakanishi et al., 2013, 2017), a concept that may be transferred to other plants, providing the possibility of an edible therapy.

Promising tools are also Stx-targeting humanized antibodies. Humanized antibodies were established by an exchange of the regions within the antigen-binding variable domains, which react with the antigen of the human IgG molecule with equivalent regions of the murine Stx-specific Mab (Nakao et al., 1999) to produce TMA-15 (Urtoxazumab) (Yamagami et al., 2001; Kimura et al., 2002). TMA-15 was shown to protect mice from a lethal challenge with STEC if given within 24 h of infection (Yamagami et al., 2001), while it was also able to reduce brain lesions and death in a gnotobiotic piglet model (Moxley et al., 2017). When tested in healthy adults or pediatric patients with a confirmed STEC infection, intravenous application of TMA-15 (Urtoxazumab) was found to be well-tolerated and safe (Lopez et al., 2010).

Chimeric murine-human MAbs (cαStx1/cαStx2) comprising the variable regions of the murine Stx1 (B-subunit) or Stx2 (A-subunit)-neutralizing antibodies 13C4 and 11E10 (Strockbine et al., 1985; Perera et al., 1988) fused to the light chain of human IgG1 were found to neutralize Stxs in mice (Bitzan et al., 2009). In addition, they were well–tolerated in healthy human volunteers when given as a single dose either separate or in combination (Dowling et al., 2005; Bitzan et al., 2009). Unfortunately, the efficacies of hybrid antibodies were often found to be lower compared to the murine parent antibodies (Tzipori et al., 2004). Another concern of chimeric MAbs is that they still retain murine IgG elements that could trigger antibody formation by treated patients. With this in mind, HuMAbs have been engineered, which encode the human heavy- and light-chain IgG genes, leading to the formation of human antibodies in response to immunization with an antigen, e.g., Stx1 or Stx2 [e.g., 5A4 (Stx1) and 5C12 (Stx2)] (Mukherjee et al., 2002a,b; Sheoran et al., 2005). These resulted in prolonged survival of mice in a Stx1 toxicosis model (Mukherjee et al., 2002b) and higher survival of gnotobiotic piglets when treated 48 h after challenge with an Stx2a-producing STEC strain (Sheoran et al., 2005). Interestingly, when piglets were infected with an Stx1- and Stx2-producing strain, only administration of 5C12 (αStx2) was protective (Jeong et al., 2010).

Another promising approach used hetero-multimeric camelid toxin-neutralizing agents containing two linked heavy-chain-only antibody V_H_ domains that neutralize Stx1 or Stx2 co-administered with an antitag MAb—an “effector Ab”—that indirectly decorates each toxin with four Ab molecules in cell-based and *in vivo* mouse models (Tremblay et al., 2013). The effector antibody binds to multiple epitope tags engineered into the VHH-based toxin-neutralizing agent. When the toxin-neutralizing agent interacts with separate sites of the Stx toxins, and each, in turn, binds to two or more effector Abs through the tags, the Stxs become decorated with sufficient Abs to prevent all symptoms of Stx1 and Stx2 intoxication and protect mice from Stx lethality (Tremblay et al., 2013). Moreover, camelid antibodies, which are special as they only contain heavy chains, have been produced that target the Stx2 B-subunit. These antibodies decreased Shiga toxicity when injected into mice and were proposed as an alternative treatment for HUS sequelae (Mejias et al., 2016). Moreover, Luz et al. have produced recombinant antibody fragments that specifically bind to and neutralize Stx2 *in vitro* (Luz et al., 2015). They further showed that mice were protected from challenge with a lethal dose of Stx2 after pre-incubation of the toxin with the antibody fragment FabC11:Stx2 (Luz et al., 2018).

A combined antibody–antibiotic (e.g., tigecycline) treatment scheme that was found to eliminate the toxicity from STEC (Skinner et al., 2015) may help to eliminate bacteria in addition to inhibiting Shiga-toxin mediated disease, decreasing the probability of transmission to others due to continued bacterial carriage and excretion.

Evaluation of the different antibody therapies against HUS, mostly in piglets and mice, showed that they mainly differ in their protective efficacy and/or their specificity to Stx variants, whereby the A-subunit specific antibodies were better neutralizers than their B-subunit specific counterparts (Tzipori et al., 2004). A very critical point for passive immunization with Stx antibodies that has to be considered for successful therapy is the time point and dosage of antibody administration. Studies using piglets or mice demonstrated that administration of the Stx2-specific HuAbs 5C12 or TMA-15 protected the animals 48 or 24 h after infection, respectively. However, when TMA-15 was used as treatment 48 h after infection, no protection was observed (Yamagami et al., 2001). This indicated that infected patients might be protected against the development of HUS when the antibodies are given shortly after the onset of diarrhea (Orth et al., 2008). However, as mice and piglets do not develop either bloody diarrhea or HUS, results describing a protective effect of Stx-specific antibodies cannot easily be transferred to humans. Moreover, knowledge about the time when the Stxs enter the bloodstream and the Stx levels in the blood and infected tissues is scarce.

### Effector- or Intimin-Targeted Antibodies

The *E. coli* secreted effector protein EspA forms the filamentous sheath of the T3SS, which aids in the transportation of the bacterial effectors into the host cells and elicits a protective immune response. A MAb (1H10) was identified to recognize the linear, conserved, and protective epitope Lys100-Val120 on the surface of EspA (Yu et al., 2011) (Figure 1B). This EspA-specific MAb was shown to inhibit EHEC-induced actin polymerization *in vitro* and conferred protection against EHEC, e.g., reduced their colonization efficiency in mice (Yu et al., 2011), which could be exploited for the development of epitope-based vaccine and MAb-based therapy. In addition, a camelid single-domain antibody (nanobody), TD4, which specifically recognized the Tir domain overlapping with the binding site of the adhesin intimin, was able to inhibit EHEC attachment and intimin-induced clustering of Tir, and reduced the colonization of EHEC on the human colonic mucosa (Ruano-Gallego et al., 2019).

### HuMAb Against Complement Component 5 (C5): Eculizumab

Eculizumab is a recombinantly produced HuMAb against the complement component 5 (C5). Binding of the antibody to C5 results in the inhibition of complement activation. Originally not devised for the treatment of STEC-induced HUS, Eculizumab was initially trialed in patients with severe STEC-HUS during the outbreak in northern Europe in 2011, as Shiga toxin had been shown to mediate complement activation (Orth et al., 2009; Morigi et al., 2011; Noris et al., 2012; Karpman and Tati, 2016) reviewed in Buelli et al. (2019), which, in turn, negatively affects renal health. Unfortunately, the results obtained for the use of Eculizumab in STEC-HUS were inconsistent. While most studies reported no benefit on renal and extrarenal outcomes (Kielstein et al., 2012; Loos et al., 2012, 2017; Menne et al., 2012), other publications reported a beneficial effect of Eculizumab treatment in pediatric cases (Lapeyraque et al., 2011) or fewer severely infected patients (Delmas et al., 2014). This indicated that the early use of Eculizumab in children with HUS may be beneficial. However, a more recent study evaluating the short and intermediate outcome of Eculizumab treatment, including 18 children with STEC-HUS in a single-center matched cohort study did not reveal a benefit of Eculizumab on renal and extrarenal outcomes (Monet-Didailler et al., 2019). It has been discussed that the delay between HUS diagnosis and Eculizumab administration could affect patient recovery (Keenswijk et al., 2017). It was also suggested that Eculizumab might improve potential neurological outcomes (Pape et al., 2015; Monet-Didailler et al., 2019).

## Toxin Receptor Analogs

The Stxs, once released from the bacterial cell, spread through the body and target cells (Lingwood et al., 1987), which express the Gb3 receptor on their cell surface, such as renal glomerular and brain endothelial cells. Binding of Stxs to the Gb3 receptors is based on the multivalent interaction of the five B-subunits with the trisaccharide moiety of Gb3. Therefore, interfering with receptor binding by using receptor analogs to probe for free toxin in the gut or the circulation is a promising approach to reduce Stx-mediated disease. Over the last decade or so, a variety of strategies have been employed to produce (i) inhibitors of the Stx receptor Gb3 to prevent Stx binding and uptake, and (ii) Stx-neutralizing Gb3 analogs (Macconnachie and Todd, 2004; Serna and Boedeker, 2008; Rahal et al., 2015; Kavaliauskiene et al., 2017) (Figure 1C).

### SYNSORB Pk

Synsorb Pk was one of the first and certainly a promising Stx receptor analog. It consists of silicon dioxide particles (diatomaceous earth) with covalently linked trisaccharides that functioned as an orally administered Stx adsorbent. SYNSORB Pk was tested in a large multicenter trial including 145 children diagnosed with STEC-induced HUS (Trachtman et al., 2003). Unfortunately, however, no treatment benefit could be observed. There were no significant differences in the number of deaths or incidence of extrarenal complications and no reduction in the need for dialysis was observed. Likely reasons for the observed failure are that (i) the agent was administered too late, (ii) the Stx toxins are mostly cell-associated and not free-floating, and (iii) the Stx binding capacity of monomeric SYNSORB Pk is substantially lower than that of Gb3 polymers.

### Starfish and Daisy

Starfish is an oligovalent, water-soluble carbohydrate ligand with a sub-nanomolar inhibitory activity designed based on the crystal structure of the Stx1 B-subunit (Kitov et al., 2000). The *in vitro* inhibitory activity is very high as the two trisaccharide receptors at the tip of a 5-spacer arm engage all five Stx1 B-subunits. Daisy is a Gb3 analog (αGal(1,4)βGal) from the same group that developed Starfish, which was shown to protect mice from Stx1- and Stx2-mediated disease by subcutaneous administration (Mulvey et al., 2003).

### SUPER TWIG (1) and (2)

Nishikawa and colleagues (Nishikawa et al., 2002, 2005) designed a series of carbosilane dendrimers carrying various numbers of terminal Gb3 moieties to bind Shiga toxin in the bloodstream before it reaches target cells expressing the receptor. The SUPER TWIG Gb3 analogs bound Stx1 and Stx2 with high affinity, prevented Stx uptake into host cells, induced phagocytosis of Stx by macrophages, and protected mice from a fatal challenge with EHEC.

### Acrylamide Polymers With Gb3 Trisaccharides

Watanabe et al. (2004) constructed acrylamide polymers of Gb3 as toxin absorbent in the gut that bound both Stx1 and Stx2 with a very high affinity [e.g., it interacted with a higher affinity to the Stx B subunit than SUPER TWIG (1)]. They further showed that the oral administration of these polymers was able to protect mice that had been orally challenged with a fatal dose of STEC, whereby the toxin content in serum samples in the treated infected mice was significantly reduced. This protection was observed even if the polymers were administered after colonization.

### Phage-Display Generated Stx-Neutralizing Peptides

Three peptides that bind to Gb3 receptor have been developed using phage-display (PC7-2, P12-26, and PC7-30). They efficiently competed with the Stx for binding and inhibited Stx-triggered cell toxicity. Peptide PC7-30 further inhibited Stx1-induced lethality in EHEC-infected animals, indicating that this peptide might be useful to prevent STEC-triggered diseases such as HUS (Bernedo-Navarro et al., 2014).

### Bacteria Expressing Gb3 Analogs

In addition to previous attempts, *E. coli* strains and probiotics can be engineered to express Gb3 receptor mimics on their surface (Paton et al., 2000; Asahara et al., 2004; Hostetter et al., 2014). They absorb and neutralize Stx1, Stx2, Stx2c, and Stx2d *in vitro* and oral administration of Gb3 analog-expressing bacteria protected mice from fatal challenge with different highly virulent STEC strains.

### Nanoparticles Displaying Stx Ligands

Kulkarni et al. (2010) established glycan-encapsulated gold nanoparticles that allowed multivalent display of glycans, e.g., the glycan Pk trisaccharide, which preferentially interacts with Stx. The coated nanoparticles neutralized Stx1 and Stx2, but not all variants in a Vero cell toxicity assay.

### Gb3 Inhibitors

Inhibitors that interfere with the synthesis of the Stx receptor Gb3 are also attractive targets. The agent C-9 is a specific inhibitor of glucosylceramide synthase that downregulates the expression of Gb3, limiting the amount of receptor displayed on the surface of cells (Silberstein et al., 2011). C-9 addition to human kidney cells in a tissue culture model decreased Stx2-mediated tissue damage. Furthermore, administration of C-9 in a rat model decreased mortality by about 50% and significantly diminished toxin-mediated tubular necrosis and damage to goblet cells. In addition, PDMP, a ceramide analog shown to inhibit the synthesis of GlcCer and affect the composition of glycosphingolipids, reduced Stx binding and uptake and blocked initial transport of the toxin into the Golgi apparatus (Raa et al., 2009). It was also discovered that the glucose analog 2-fluoro-2-deoxy-D-glucose (FDG) reduced cellular uptake of Gb3 levels by 50%, likely by inhibiting precursor formation of Gb3 synthesis, and resulted in a decrease in Stx binding (Kavaliauskiene et al., 2015, 2016).

### Intracellular Interference With Shiga Toxins

Not only Stx binding and uptake, but also its intracellular targeting from early endosomes to the Golgi apparatus and the endoplasmic reticulum (ER) can be inhibited by certain cell-permeable agents. One such substance is chloroquine, a weak base that can diffuse across membranes and accumulate in acidic compartments. Chloroquine treatment protected HEp-2 cells from Stx-mediated cytotoxicity, most likely by interfering with the translocation of the StxA subunit into the cytosol (Dyve Lingelem et al., 2012; Kavaliauskiene et al., 2017). The Retro-1 and−2 substances were shown to inhibit the retrograde transport of Stx1B from the endosomes to the Golgi apparatus, and it has been assumed that this could be mediated by the relocalization of the SNARE proteins syntaxin 5 and 6 (Noel et al., 2013; Kavaliauskiene et al., 2017). Later, it has been shown by Secher et al. (2015) that Retro-2 was able to protect mice against the toxic effects of Stx. Recently, a drug delivery system using Retro-2-loaded nanoglobules has been developed, which increased the solubility of the inhibitor and might enable a more successful therapeutic approach inhibiting the transport of Stx (Gandhi et al., 2019). Moreover, two other substances, Ac-PPP-tet and TVP, which also interfere with intracellular Stx trafficking, have been tested in animal models and were found to successfully prevent Stx2 intoxication (Watanabe-Takahashi et al., 2010; Stearns-Kurosawa et al., 2011).

## Vaccination Strategies

### Toxin-Based Vaccines

**Figure 2:**
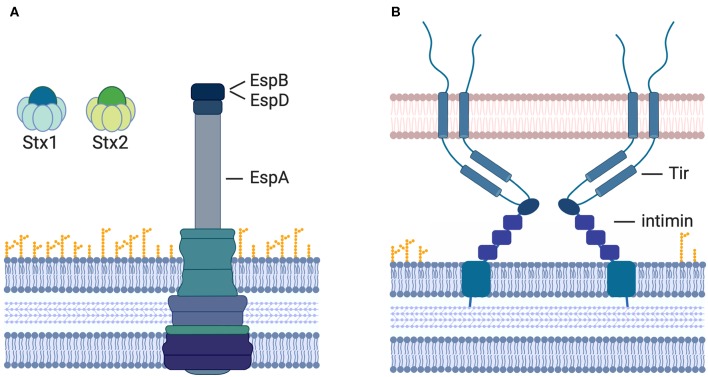
Vaccination strategies to prevent STEC-induced diseases. Currently assessed vaccine targets include **(A)** the A-and B-subunits of Stx1 and Stx2, the T3SS components EspA (sheath) and EspB (pore complex), as well as **(B)** the bacterial outer membrane protein intimin and its T3-translocated receptor Tir.

Stx is the main virulence factor associated with the potency of STEC-mediated disease pathology. It is released from the bacterial cell and exposed to the host immune system and has, therefore, long been considered one of the most prudent targets for vaccine strategy. Once released from the bacterial cell, Stx can be detected in the intestinal lumen and its target cells in the kidney and brain (Clements et al., 2012). Hence, immune cells primed to recognize Stx by prior vaccination can interfere and respond to the toxin as it travels from the site of release to the distal organs and eliminate it before it reaches its targets (Figure 2A). Of Stx1, Stx2, and the different varieties of each of these subtypes, Stx2 has been at the center of a greater number of vaccination approaches as it is commonly associated with more severe disease outcomes in humans, such as the development of HUS. Several vaccination strategies have been developed and tested that are solely toxin-based. With the active site of Stx known, inactive derivatives of the toxin are easy to make, and present safe alternatives for application. Several different studies showed that vaccination of animals with purified inactive Stx derivatives induced the production of neutralizing antibodies against the respective toxin and protected the animals from toxemia or limited shedding or disease after challenge (Gordon et al., 1992; Acheson et al., 1996; Konadu et al., 1999; Marcato et al., 2001, 2005; Ishikawa et al., 2003; Kerner et al., 2015; Schmidt et al., 2018). In addition to vaccines based on the inactive toxin, hybrid subunit vaccine approaches have been tested as the development of a vaccine, which may induce neutralizing antibodies against not only one but both types of Stx, would be ideal.

Vaccination with a hybrid toxin consisting of an inactive Stx2 A-subunit fused to the native Stx1 B-subunit was able to produce neutralizing antibodies against both Stx1 and Stx2. Mice immunized with this toxin were protected from subsequent lethal challenge with either Stx1 or Stx2, or both toxins (Smith et al., 2006).

Injection with a purified fusion protein consisting of the B-subunits of Stx subtypes 1 and 2 (Stx2B-Stx1B; short 2S) generated neutralizing antibodies against both Stxs and increased survival of mice after a challenge with *E. coli* O157:H7 lysates (Gao et al., 2009). Interestingly, the protective effects of the vaccine were stronger with the Stx2B-Stx1B-subunit fusion than when separate B-subunits were used for immunization (Gao et al., 2009).

Moreover, a fusion protein comprising the B-subunit of Stx1 and the inactive A-subunit of Stx2 (Stx2Am-Stx1) was constructed. Immunization with this protein resulted in a strong induction of neutralizing antibodies against both types of Shiga toxin and increased the survival rate of mice after challenge with *E. coli* O157:H7 lysates (Cai et al., 2011). A vector-based DNA vaccine encoding the C-terminal 32 amino acids of the Stx2 A-subunit and the complete B subunit (pStx2ΔAB) also produced neutralizing antibodies against both Stx2 subunits. It further decreased the mortality of mice after lethal challenge with Stx2 (Bentancor et al., 2009).

Intranasal immunization with the Stx2 B-subunit in combination with a mutant of heat-labile toxin induced neutralizing antibodies against both Stx1 and Stx2 *in vivo* and protected mice against fatal disease. Interestingly, immunization of mice with the B-subunit of Stx1 only protected against subsequent challenge with Stx1 (Tsuji et al., 2008). A fusion of the Stx B-subunit to the B-subunit of heat-labile toxin has also been assessed for toxin subtype 2e. Here, the ability of the fusion protein to induce neutralizing antibody production was much higher than for the Stx2 B-subunit alone. Furthermore, mice that had been immunized with the Stx2eB-LTB fusion protein were protected against challenge with a lethal dose of toxin (Ran et al., 2008).

In another study, the B subunit of Stx2 was fused with *Brucella* lumazine synthase, a protein that forms a dimer of pentamers thereby creating a scaffold for the presentation of the Stx. The resulting fusion protein induced a lasting immune response in mice after three vaccinations and vaccinated mice were protected from intravenous challenge with Stx2. Furthermore, antibodies isolated from vaccinated mice neutralized Stx2 as well as its variants. In addition, weaned mice inoculated with the immune sera were protected against oral infection with EHEC (Mejias et al., 2013).

### Vaccine Approaches Based on LEE-Encoded Proteins

While Stx is produced by all strains that are classed as STEC, only some STEC strains encode the locus of enterocyte effacement pathogenicity island (LEE), which encodes for the T3SS. Therefore, the use of T3-secreted proteins in vaccine approaches is valid but limits the specificity of the vaccines to LEE-positive STEC strains. As the most common EHEC strains including those of the O157:H7 serotype are LEE-positive, the T3-S protein-based approaches will target many of the most common serotypes. However, they do not affect other pathotypes such as the O104:H4 strain, which caused the outbreak in Germany in 2011.

Only a few of the T3-secreted proteins can be used as targets for vaccination strategies, however, as most are translocated from the bacterial cell directly into the host cell cytoplasm and are never exposed to the surrounding environment. A few proteins including EspA, EspB (Figure 2A), Tir, and intimin (Figure 2B) are exposed on the outside of the cell at times as antibodies to these proteins are detectable in humans after an EHEC infection (Li et al., 2000; Asper et al., 2011). These proteins have been assessed as targets for vaccination strategies. The translocated intimin receptor (Tir) inserts into the host cell membrane upon translocation. Its surface-exposed receptor domain then interacts with the bacterial outer membrane protein intimin to induce intimate attachment of the bacteria to the host cell surface.

Furthermore, protein components of the T3 secretion system such as the sheath protein EspA, as well as EspB and EspD, the proteins required for forming a pore in the host cell membrane, have also been assessed for their immunogenicity (Loureiro et al., 1998; Martinez et al., 1999; Asper et al., 2011; Guirro et al., 2013). Most T3-secreted protein-based vaccines have been assessed using the intranasal immunization route. This vaccination approach promises a needle-free application and has, so far, yielded promising results.

Subcutaneous and intranasal immunization of mice with T3-secreted proteins showed that while subcutaneous injection was unable to raise an immune response, intranasal vaccination induced the production of anti-Tir and EspA antibodies. This reduced *E. coli* O157:H7 shedding after infection (Babiuk et al., 2008). Subcutaneous and intranasal immunization with purified Tir showed similar results. Mice immunized intranasally produced neutralizing antibodies, which resulted in reduced fecal shedding of *E. coli* O157:H7 after infection and increased animal survival (Fan et al., 2012). Intranasal immunization of mice with a purified fusion protein consisting of EspB and the C-terminus of intimin induced neutralizing antibodies against both EspB and intimin and antisera of immunized mice had promising anti-hemolytic effects *in vitro* (Cataldi et al., 2008).

A recombinant fusion protein of EspA, intimin, and Tir (EIT) was created and used for immunization of mice. Subcutaneous or oral immunization of mice with the EIT protein resulted in a significant decrease of bacterial colonization and shedding after challenge with EHEC O157:H7 and an increase in anti-EIT IgG and IgA (Amani et al., 2010). In a follow-up study, rEIT was linked to chitosan and used for either intranasal electrospray (Doavi et al., 2016) or oral (Khanifar et al., 2019a) immunization of mice. Intranasal and oral administration both induced specific immune responses and reduced bacterial shedding after challenge with *E. coli* O157:H7 (Doavi et al., 2016). Oral administration additionally helped protect mice against *E. coli* O157 challenge and reduced damage (Khanifar et al., 2019a). An additional approach was made by encapsulating EIT together with the B-subunit of Stx2 (Khanifar et al., 2019b). Mice were subcutaneously or orally immunized and either infected with *E. coli* O157:H7 or challenged with a fatal dose of Stx2. While the former mice showed reduced colonization and bacterial shedding, the latter showed increased survival (Khanifar et al., 2019b). A shortened variant of the EIT fusion protein consisting of only EspA and intimin (EI) was recombinantly expressed and its immunogenicity was assessed after subcutaneous injection with two subcutaneous boosters and a third booster that was administered i.p. (Rad et al., 2013). This fusion protein, too, induced an immune response and decreased bacterial shedding and histopathological changes in the intestine after challenge (Rad et al., 2013).

In addition, the immunogenicity and protective efficacy of a DNA vaccine against a truncated version of the EHEC factor for adherence-1 (Efa-1'; the homolog of LifA in EPEC and *C. rodentium*) was evaluated in mice. Intranasal immunization with plasmid DNA induced efa-1-specific immune responses and protected mice from subsequent challenge with *E. coli* O157:H7 (Riquelme-Neira et al., 2015).

Peptide-based approaches to vaccination include the KT-12 peptide, which is based on a predicted B-cell epitope of intimin conjugated to adjuvant (Wan et al., 2011) and the synthetic peptides CoilA and CoilB, which interact with EspA (Larzabal et al., 2013). KT-12, when used for intranasal immunization, induced the production of neutralizing antibodies and protected mice from challenge with *E. coli* O157:H7 (Wan et al., 2011). Immunization of mice with CoilA and CoilB was shown to block intestinal damage in mice infected with *C. rodentium* (Larzabal et al., 2013).

A fusion of EspA, the C-terminus of intimin and the B-subunit of Stx2 (EIS), was constructed and assessed for its ability to induce the production of neutralizing antibodies. Indeed, antibodies against all three components of the fusion protein were detected, and immunized mice were protected from challenge with *E. coli* O157:H7 or lysates thereof (Gu et al., 2009). Fusion of the processed, active form of the Stx2A-subunit (Stx2A1) to the N-terminus of EspA induced the production of neutralizing antibodies in immunized mice (Cheng et al., 2009) and a fusion of the B-subunits of Stx1, Stx2, to a truncated version of intimin resulted in increased immune responses and protection of mice after a fatal challenge with *E. coli* O157:H7 (Gao et al., 2011).

Intranasal immunization with a novel EspA-Tir fusion protein (EspA-Tir-M; designating that the middle domain of Tir was used) showed high levels of neutralizing antibodies while subcutaneous vaccination had little effect. Additionally, intranasal immunization increased the survival of mice from subsequent challenge with *E. coli* O157:H7 and reduced organ damage (Lin et al., 2017b).

## Bacteria-Based Vaccines

### Attenuated or Vaccine Strains

Several vaccination approaches use non-pathogenic bacteria or bacterial vaccine strains as delivery vehicles and to increase immunogenicity. These approaches include genetically modified EHEC, EPEC, and *Salmonella* strains as well as probiotic strains such as *Lactococcus lactis* and *Lactobacillus acidophilus*.

A non-pathogenic variant of the EHEC O157:H7 86-24 strain was created by deletion of both the gene encoding the transcriptional regulator of the LEE (*ler*) and the *stx* gene. These deletions completely abolished cytotoxicity *in vitro* when compared to EHEC EDL933. A derivative of this strain that expresses the inactive forms of Stx1 and Stx2 from a plasmid also showed highly diminished cytotoxicity *in vitro*. Injection with either *stx/ler* deletion mutant or the respective Stx1/Stx2-expressing strain reduced the colonization of *E. coli* O157:H7 after infection of mice. Furthermore, if mice were immunized when pregnant, they passed the immunity on to their offspring, which were protected against *E. coli* O157:H7 infection (Liu et al., 2009).

Enteropathogenic *E. coli* (EPEC) also presents a vaccine alternative for EHEC as it is less pathogenic and shares the LEE pathogenicity island-encoded virulence genes. Immunization with EPEC raised neutralizing antibodies against EspB and intimin and conferred some protection against an EHEC infection. Vaccinated mice showed only mild disease phenotypes such as slight intestinal damage, while no kidney pathology could be detected (Calderon Toledo et al., 2011).

Immunization of mice with a *Salmonella* Typhimurium vaccine strain expressing an inactive Stx2 variant consisting of the A2- and B-subunit of Stx2 (Stx2ΔAB) resulted in efficient colonization of the Peyer's patches and production of neutralizing antibodies. Serum collected from immunized mice was able to neutralize Stx2-mediated toxicity *in vitro*. However, there was only minimal protection observed when mice were challenged with a lethal dose of Stx2, and no protective effect was seen for kidney health (Rojas et al., 2010).

Additionally, another group constructed a *Salmonella* Typhimurium strain expressing intimin, which was used to immunize mice orally (Oliveira et al., 2012). This immunization resulted in a significant increase in the levels of serum IgG and fecal IgA and reduced fecal shedding after an *E. coli* O157:H7 infection (Oliveira et al., 2012). A boost vaccination 2 weeks after the initial immunization led to continuously high colonization levels of the vaccine strain and dissemination into the underlying tissues such as Peyer's patches and spleen (Oliveira et al., 2012). Oral immunization of mice with attenuated *Salmonella* expressing a hybrid protein consisting of EspA in combination with the C-terminus of intimin and the Stx2 B-subunit (EIS, also see above) raised neutralizing antibodies against the respective proteins and protected mice from a lethal challenge with EHEC for more than 70 days. This period could be extended by a subcutaneous boost with purified EIS (Gu et al., 2011).

Inoculation of mice with a recombinant *Mycobacterium bovis* BCG (rBCG) vaccine, which was modified to express the Stx2 B-subunit, induced the production of neutralizing antibodies against Stx2. Two high-dose intraperitoneal immunizations resulted in decreased colonization and increased survival after fatal challenge with a STEC strain (Fujii et al., 2012).

The probiotic lactic acid bacterium *Lactococcus lactis* is considered a safe vaccine vehicle. Use of a *L. lactis* strain expressing the Stx2 A1-subunit (the A-subunit missing the 15 C-terminal amino acids) for the immunization of mice resulted in increased levels of fecal and serum IgA. Immunized animals had significantly reduced intestinal and kidney damage. Furthermore, immunized mice showed increased survival after challenge with a lethal dose of Shiga toxin isolated from either *E. coli* O157:H7 or *Shigella dysenteriae* (Sreerohini et al., 2019).

*L. lactis* expressing the T3-secreted protein EspB did not yield neutralizing antibodies when used to infect mice. After an i.p. boost with recombinant EspB, however, specific IgG and IgA levels increased (Ahmed et al., 2013). In a follow-up study, the *L. lactis* was modified to secrete EspB after expression, which resulted in an increased production of neutralizing antibodies. Also, mice immunized with this version of the EspB-expressing *L. lactis* were protected against *E. coli* O157:H7 colonization (Ahmed et al., 2014). An *L. lactis* strain expressing the EspA protein has also been designed. However, this strain has so far only been used for the production of recombinant EspA, as described above. Here, too, a system to either display the protein at the cell surface or secrete it from the cell will probably be needed but may be worthwhile (Luan et al., 2010). A recombinant *L. lactis* strain that displays the Stx1 B-subunit via albumin binding domains (single-domain non-immunoglobulin scaffolds) on the bacterial cell surface was recently designed by Zadravec et al. (2016). ELISA and FACS analysis confirmed the ability of this strain to bind Stx1. The immunogenicity and safety of this strain and its ability to protect against challenge were, however, not yet tested in animals.

Lastly, a recombinant *Lactobacillus acidophilus* variant expressing EspA and the Tir central domain (EspA-Tir-M) inhibited A/E lesions formation by EHEC O157:H7 after pre-incubation *in vitro*. Oral immunization of mice induced the production of specific and systemic neutralizing antibodies and reduced EHEC O157:H7 colonization. It also inhibited intestinal A/E lesions and toxin-mediated organ damage (Lin et al., 2017a).

### Bacterial Ghosts

Bacterial ghosts (BGs) remain when bacteria are treated with viral E protein. This protein forms tubes across the bacterial cell membrane releasing the cytoplasm of the bacteria into the surroundings. What remains are bacterial ghosts, empty membranes with intact bacterial morphology and cell surface structures. Because of this, bacterial ghosts are highly immunogenic. Moreover, due to the ubiquitous process used to create them, bacterial ghosts can be produced from whichever strain is desirable (Lubitz et al., 2009; Hajam et al., 2017).

*E. coli* O157:H7 N°CIP 105282 encode Stx1 and Stx2. BGs were produced from this strain by combining treatment with viral E protein to remove the cytoplasm and addition of staphylococcal nuclease A to degrade pathogenic DNA. Oral immunization of mice induced a specific immune response and mice were protected from a subsequent challenge with an EHEC strain (Mayr et al., 2005). When oral immunization was followed by an oral boost on day 28, antibody production increased, resulting in even better survival (Mayr et al., 2005). A later study showed that a single rectal inoculation of mice with these BGs led to the production of neutralizing antibodies that completely protected mice from infection with a lethal dose of bacteria without requiring a boost (Mayr et al., 2012).

The use of *E. coli* O157:H7 (EDL933) bacterial ghosts yielded similar results. Here, too, an increase in neutralizing antibodies and protection was observed after a boost (Cai et al., 2010). When the BGs were modified to display an inactive Stx2A–Stx1B fusion (Stx2Am–Stx1B) on the cell surface, a stronger induction of neutralizing antibodies was observed, which correlated with better survival and reduced organ damage upon challenge with EHEC. Furthermore, bacterial ghosts displaying the Stx2Am–Stx1B on the surface performed better than those that did not, suggesting that the combination of surface antigens such as intimin in combination with the toxin resulted in even better immune responses (Cai et al., 2015).

### Outer Membrane Vesicles

Outer membrane vesicles (OMVs) are nanoparticles that are released by many Gram-negative bacteria including *E. coli*. Their major component is bacterial LPS, making them highly immunogenic. Modified OMVs from Shiga toxin A-subunit-deficient *E. coli* O157:H7 were prepared and tested by eyedrop application for their activity against HUS development. Mice received a boost after 2 weeks, and subsequent intraperitoneal challenge with wild-type OMVs was carried out 4 weeks after the initial immunization. The vaccinated mice were shown to be protected from the lethality usually observed upon challenge with wildtype OMVs (Choi et al., 2014). Immunization with chemically inactivated OMVs obtained from a virulent *E. coli* O157 strain was also effective in the murine infection model. Here, mice were immunized subcutaneously on days 0 and 21 and received an intraperitoneal challenge with concentrated cell supernatants 2 weeks after application of second immunization. While 90% of control mice died by day seven post-challenge, all immunized mice survived (Fingermann et al., 2018).

### Plant-Based Vaccines

Plant-based vaccines have been trialed with the idea that they can easily be used for oral vaccination.

#### Toxin-Targeted Plant-Based Vaccines

The inactive A-subunit of Stx2 was produced by expression in a tobacco plant cell line (NT-1). Subsequent immunization of mice by feeding or by parenteral immunization with an oral boost resulted in increased Stx2 IgA and IgG levels. It was able to protect mice from a lethal challenge with an EHEC strain. Sera of immunized mice further neutralized toxicity *in vitro* (Wen et al., 2006). For the treatment of porcine edema disease, which is a severe and often fatal disease in pigs that is also mediated by Stx (in this case subtype Stx2e), Hamabata et al. have recently developed *stx2eB*-transgenic lettuce for immunization. Infection of piglets with STEC after oral vaccination by feeding the lettuce showed decreased levels of pathogenesis in lettuce-fed piglets (Hamabata et al., 2019).

#### Virulence Protein-Targeted Vaccines

The immunization capacity of plant-codon optimized intimin expressed in the NT-1 tobacco cell line was also assessed by either i.p. injection of purified protein, feeding of transgenic plant cells, or a combination thereof. Here, mice immunized by injection and boosted by feeding developed neutralizing antibodies against intimin and reduced the time of bacterial shedding after challenge with *E. coli* O157:H7 (Judge et al., 2004). In another approach, chimeric protein composed of the LEE-encoded proteins EspA, intimin, and Tir (named EIT and further described above) was codon-optimized for expression in tobacco and canola plants. Using this plant-based expression strategy, recombinant EIT was prepared, and immune responses in mice were assessed after parenteral and oral immunization as well as after a combination. Here, a combination of subcutaneous injection and oral gavage yielded the highest immune responses and resulted in significantly reduced fecal shedding of *E. coli* O157:H7 after challenge (Amani et al., 2011).

## Inhibitors

### Pyocins

R(rod)-type pyocins are bacterial structures that have developed from phages and show a high similarity to bacteriophage tails. R-type pyocins bind to the core polysaccharides in the outer membrane of bacteria via specific receptor binding proteins, resulting in selective targeting of certain bacteria. The LPS recognition induces contraction of the phage-like tail and puncturing of the bacterial cell envelope, leading to loss of bacterial membrane potential and subsequent cell death. This mechanism is best described for *Pseudomonas aeruginosa*, which use pyocins to target competitors. However, by designing hybrid proteins that possess the receptor binding proteins or tail fibers of known bacteriophages, the specificity of the pyocins can be changed (Kim et al., 2019). An EHEC O157:H7 specific pyocin (AvR2-V10) developed by Scholl et al. was able to specifically recognize and degrade O157 LPS and kill *E. coli* O157:H7 without inducing Stx expression (Scholl et al., 2009). A new version of this pyocin (AvR2-10.3) was tested *in vivo* in a rabbit EHEC infection model. Here, reduced diarrhea colonization and bacterial shedding, as well as less intestinal damage, were observed (Ritchie et al., 2011). Furthermore, Scholl et al. identified an O104-specific bacteriophage tail protein and showed that fusion of this protein to the pyocin tail fiber resulted in specific targeting and killing of O104 strains by the pyocin (AvR2-104.1) (Scholl et al., 2012), showing that this approach is promising for a variety of different serotypes.

## Alternative Approaches Using Antimicrobial Agents

### Probiotics

Management of STEC-mediated diseases including HUS onset (curative or preventive) by probiotics has also recently been addressed in many studies. The administration of certain probiotics to humans or reservoir animals may reduce colonization and carriage of STEC, which will prevent and/or reduce the risk of infection and transmission of the pathogenic bacteria (Sargeant et al., 2007; Corr et al., 2009). Protective and beneficial capabilities of probiotics have been described in several studies in which probiotics have been applied prior to an STEC infection of cultured cells or in mice (recently reviewed in detail by (Eaton et al., 2011; Mogna et al., 2012; Chen et al., 2013; Kakisu et al., 2013; Rund et al., 2013; Cordonnier et al., 2017; Giordano et al., 2019)). Significant inhibitory effects against the growth of STEC have been demonstrated for several *Lactobacillus* strains (*L. rhamnosus* LR04, LR06, *L. delbrueckii, L. pentosus, L. fermentum, L. crispatus, L. plantarum, L. lactis, L. kefir, L. gasseri* CCDM215*, L. accidophila* CCDM149*, L. helveticus* KLDS, *L. casei, L. paracasei* NZU101), *E. coli* Nissle, *Enterococcus faecium* YF5, *Enterococcus faecalis* (Symbioflor), *Bifidobacterium longum*, and others. Moreover, use of direct-fed microbiota was found to reduce shedding of *E. coli* O157:H7 in cattle (Peterson et al., 2007; Callaway et al., 2009; Rahal et al., 2015; Wisener et al., 2015; Giordano et al., 2019). The success seems to depend on the probiotic strain(s), immunomodulation of the host, and their metabolism and ability to modify the local milieu, e.g., by the production of short chain acids, such as lactate, butyrate, and acetate (Ogawa et al., 2001; Takahashi et al., 2004; Carey et al., 2008; Fukuda et al., 2011), or/and occupy similar niches in the intestinal tract in which they compete for adhesion to the gut epithelium and nutrients. In fact, recent studies demonstrated that the efficiency of colonization of the different gut section by intestinal pathogens is dependent on the composition of the microbiota (Baumler and Sperandio, 2016; Litvak et al., 2019).

Despite the observed beneficial effects in animals, it is very difficult to extrapolate the data to humans, considering that the beneficial ratio of probiotic to pathogen varied from 1:1 to 1:10^5^ colony forming units (CFU). Furthermore, it remains unclear how and when the probiotic should be administered.

### Phages

Another upcoming potential measure to prevent infectious bacterial diseases is phage therapy, i.e., the application of lytic phages to kill and decrease the number of pathogens in food, animal reservoirs, or patients. One of the first attempts to eliminate STEC from animals (e.g., mice, sheep, and cattle) and food was performed with bacteriophages (e.g., the T-even bacteriophage CEV1, rV5, WV8, WV7, wV11, e11/2, and e4/1c), which have the potential to lyse *E. coli* O:157:H7 (Raya et al., 2006; Sheng et al., 2006; Abuladze et al., 2008; Rivas et al., 2010; Stanford et al., 2010). To date, a large variety of phages have been isolated and shown to be highly effective in the killing of STEC strains *in vitro*, but the application of individual bacteriophages *in vivo* seemed less promising, likely due to the fact that bacterial access of the phages in the gut is reduced, or the intestinal environment is disadvantageous for phage survival and/or replication (Niu et al., 2009; Dini et al., 2016; Sabouri et al., 2017; Wang et al., 2017; Safwat-Mohamed et al., 2018). However, more recently, use of certain phages and phage cocktails including multiple STEC-specific phages for oral or rectal administration to ruminants or for spraying on fruits and vegetables has shown the potential of phage therapy to reduce STEC carriage in domestic animals (Niu et al., 2009; Dini et al., 2016; Sabouri et al., 2017; Wang et al., 2017; Safwat-Mohamed et al., 2018).

### Antibiotics

Early studies investigating the effect of antibiotics in the treatment of STEC infections have suggested that antibiotics induce the bacterial SOS response, resulting in an increase of Shiga toxin production and release (O'Brien et al., 1984; Muhldorfer et al., 1996; Kimmitt et al., 2000). This has raised concerns that antibiotics may increase the risk of HUS development (Zimmerhackl, 2000; Panos et al., 2006; Kakoullis et al., 2019), and therefore, their use during STEC infections has been contraindicated. However, not all studies were able to confirm Stx induction or an increase in the amount of HUS incidences in response to antibiotics. Furthermore, the effects of antibiotics on *stx* expression vary greatly and are dependent on the antibiotic class, the antibiotic concentration, the respective STEC strain, as well as the Stx subtype (Walterspiel et al., 1992; Grif et al., 1998; Kimmitt et al., 2000; Ochoa et al., 2007; Pedersen et al., 2008; Zhang et al., 2009; McGannon et al., 2010; Bielaszewska et al., 2012; Nassar et al., 2013). While the results obtained from some antibiotic classes, such as β-lactams, are conflicting (Yoh et al., 1997, 1999; Grif et al., 1998; Yoshimura et al., 1999; McGannon et al., 2010; Muhlen et al., 2020), ansamycins and chloramphenicol consistently yielded promising results in *in vitro* studies (Kimmitt et al., 2000; Ochoa et al., 2007; McGannon et al., 2010; Kakoullis et al., 2019; Muhlen et al., 2020) while fluoroquinolones were regularly associated with toxin induction (Zhang et al., 2000; Hiramatsu et al., 2003; Bielaszewska et al., 2012; Berger et al., 2019; Muhlen et al., 2020).

Some animal studies used to mimic STEC- or Stx-mediated disease in response to antibiotic treatment confirmed previous *in vitro* results, while others were contradicting the *in vitro* data (Zhang et al., 2000, 2009; Rahal et al., 2011a; Zangari et al., 2014; Kakoullis et al., 2019). Interestingly, the *in vivo* studies confirmed a detrimental effect of fluoroquinolone antibiotics (Zhang et al., 2000; Hiramatsu et al., 2003; Muhlen et al., 2020) and a reduction of disease pathology when animals were treated with rifamycin antibiotics (Rahal et al., 2011a,b; Muhlen et al., 2020).

As studies using antibiotics that inhibit transcription or translation, such as rifamycins, have regularly been shown to inhibit Stx production *in vitro* and *in vivo* (Rahal et al., 2011a,b; Corogeanu et al., 2012; Fadlallah et al., 2015; Berger et al., 2019; Muhlen et al., 2020) even after pre-treatment with fluoroquinolone ciprofloxacin (Berger et al., 2019), this opens up the possibility of a potential treatment regimen for STEC infection using antibiotics combination therapy consisting of a transcriptional inhibitor supplied in advance of or simultaneously with an antibiotic such as ciprofloxacin, which efficiently clears the bacterial infection.

### Natural Products

Several studies have tested natural products for their ability to reduce or prevent Stx-induced cell or tissue damage in cell cultures *in vitro* or in animal infection models. An inhibition of cytotoxicity of Stx of *E. coli* O:157:H7 was found for white carob tree (*Prosopsis alba*) and *Ziziphus mistol* extracts (Pellarin et al., 2013) and for Ellagitannin from the Aleppo oak (*Quercus infectoria*) (Voravuthikunchai et al., 2012). Moreover, bacterial products such as lactic acid, linoleic acid, (Pittman et al., 2012), green tea extracts (Isogai et al., 1998, 2001), fruit juices (Nogueira et al., 2003), and other plant products (Takemasa et al., 2009; Lacombe et al., 2010; Lee and Stein, 2011; Sheng et al., 2016; Sewlikar and D'Souza, 2017; Patel et al., 2018) have been shown to have a beneficial effect on STEC-infected cells and animals alone or in combination of other agents, e.g., antibiotics.

## Conclusions

Although almost four decades have passed since the first clinical human HUS case (Centers for Disease Control, 1982) and the incidence of HUS increases, a generally accepted and successful therapy for STEC-induced HUS in patients is still missing. Nevertheless, a number of promising approaches and clinical studies employing different strategies have been performed. Here, we have reviewed therapeutic strategies including Stx toxin receptor analogs, Stx-specific antibodies, and alternative antimicrobial agents. For some, a beneficial effect has been reported, although the outcome seems to depend on multiple factors, e.g., the dose of the agent, the STEC isolate/strain, the Stx variants, the time point of administration, the route of application, the severity of the infection/symptoms, and the type of agent. Another important problem is that our information about the concentration, activity, and the precise localization of the toxins during the course of the infection is still scarce. Further studies examining these issues and testing the efficacy of Stx-inhibiting agents according to gained knowledge from these experiments are required to select the most successful regimens that could then be assessed in clinical trials. As none of the current animal models mimic EHEC infections and HUS development in humans, clinical trials and cohort studies are urgently needed to evaluate whether newly developed treatment strategies are effective. In this context, it should also be mentioned that early detection of an EHEC infection is crucial for the success of most newly developed therapeutics. Thus, the development of fast and reliable diagnostic tools to screen for Stx is also of utter importance. Moreover, a vaccination strategy for humans, but also for cattle limiting STEC carriage in their reservoirs would be of great benefit for public health.

What is the current situation of possible therapies and vaccines? While many different treatment strategies have been employed in order to develop a therapeutic or prophylactic treatment against STEC-induced disease, few have, to date, progressed past Phase II trials. Several approaches including monoclonal antibodies, receptor analogs such as Synsorb Pk, or the use of Eculizumab looked promising, but when evaluated systematically or in Phase III trials showed little evidence of success. The disadvantage of monomeric antibodies may likely be that the Stx receptor forms polymers, thus leaving Stx docking spaces available. Newly developed antibodies and neutralizing peptides therefore aim at providing multimeric recognition sites to capture as many receptor-binding sites as possible. These have, however, yet to be tested in clinical trials.

Vaccine approaches face other difficulties. While STEC infections are an important cause for diarrhea disease and especially dangerous as they can lead to HUS and systemic complications, the total amount of STEC infections is rather low. Therefore, a prophylactic vaccine may only be of interest for countries in which these infections are endemic. In general, while phase II clinical trials can be carried out, the availability of patients with STEC infections for phase III trials is limited. Furthermore, these infections will vary in the infection-causing strain and Stx subtype, both of which need to be controlled in a clinical trial. Also, the time of presentation at the physician or in hospital will most likely be after the onset of bloody diarrhea or late stages of watery diarrhea, making an early intervention difficult.

## Author Contributions

All authors listed have made an equal, substantial, direct and intellectual contribution to the work, and approved it for publication.

### Conflict of Interest

The authors declare that the research was conducted in the absence of any commercial or financial relationships that could be construed as a potential conflict of interest.
